# Adult WHO grade II ependymomas: is Ki67 a sex-specific proliferation marker?

**DOI:** 10.1093/jscr/rjab333

**Published:** 2021-08-24

**Authors:** Asfand Baig Mirza, José Pedro Lavrador, Marco Mancuso-Marcello, Shami Acharya, Timothy Martyn Boardman, Istvan Bodi, Richard Gullan, Francesco Vergani, Ranj Bhangoo, Keyoumars Ashkan

**Affiliations:** Department of Neurosurgery, King’s College Hospital NHS Foundation Trust, Denmark Hill, London, UK; Department of Neurosurgery, King’s College Hospital NHS Foundation Trust, Denmark Hill, London, UK; Department of Neurosurgery, King’s College Hospital NHS Foundation Trust, Denmark Hill, London, UK; Department of Neurosurgery, King’s College Hospital NHS Foundation Trust, Denmark Hill, London, UK; GKT School of Medical Education, King’s College London, London, UK; Department of Neuropathology, King’s College Hospital NHS Foundation Trust, Denmark Hill, London, UK; Department of Neurosurgery, King’s College Hospital NHS Foundation Trust, Denmark Hill, London, UK; Department of Neurosurgery, King’s College Hospital NHS Foundation Trust, Denmark Hill, London, UK; Department of Neurosurgery, King’s College Hospital NHS Foundation Trust, Denmark Hill, London, UK; Department of Neurosurgery, King’s College Hospital NHS Foundation Trust, Denmark Hill, London, UK

## Abstract

Ki67 is a marker for proliferation of a given cell population. Low expression of Ki67 may be associated with a favourable outcome. We investigate how the proliferation index correlates with the location, morphology and behaviour of WHO grade II ependymomas with a single-centre cohort study of adult patients admitted for surgery of WHO grade II ependymomas between 2008 and 2018.

Seventeen patients were included, seven had supratentorial and 10 had infratentorial tumours. Three patients died and eight had recurrent disease. Age, gender, location, extent of resection, chemotherapy, radiotherapy and histological markers were not associated with tumour progression. Both unadjusted and adjusted analysis confirmed a higher Ki67 index in male patients. Sensitivity analysis further supported the correlation between Ki67 and male gender.

Ki67 may be sex specific but does not seem to correlate with survival and time to recurrence in this series.

## INTRODUCTION

Ependymomas are the most common type of spinal cord tumours, however are relatively rare neoplasms of the brain, especially in adults, comprise 3% of all primary central nervous system (CNS) tumours diagnosed annually in the USA [1].

Classically, the literature distinguishes types of intracranial ependymomas by (i) ‘Location’; whether they are supra- or infratentorial, (ii) ‘Morphological Phenotype’; e.g. cellular, papillary, tanycytic, clear cell, pigmented, epithelioid or giant cell and (iii) ‘traditional WHO grading’ [2], grade I is a subependymoma or myxopapillary ependymoma, grade II is a classical ependymoma and grade III is an anaplastic ependymoma. However, the clinical value of location, histological grade and morphology is controversial given the heterogeneity of the case series in the literature [3].

DNA methylation profile is now thought to be a more informative lens with which to view and understand lesions [4]. In 2016 the updated WHO Classification for CNS tumours added a layer of molecular categorization for tumours for the first time [5]. A new category of ependymomas was added in the form of the RELA fusion–positive ependymoma that account for most of supratentorial ependymomas in children.

**Table 1:** Demographic characteristics

		Supratentorial	Infratentorial
Gender Male Female	9 8		
Age (years) Location	51.29 ± 3.91		
Supratentorial Infratentorial	7 10		
Number of surgeries 1 2 3		4 2 1	7 3 −
Extent of resection (first surgery) Bx STR GTR		2 3 2	- 7 3
Extent of resection (second surgery) Bx STR GTR		− 1 2	− 1 1
Radiotherapy		3	4
Chemotherapy		2	−
Follow-up (months)		67.76 ± 10.53	

Bx, biopsy; STR, subtotal resection; GTR, gross total resection.

The management of ependymomas by histological grade has not been standardized but surgical resection and radiotherapy are the mainstay of treatment with chemotherapy being of little efficacy [6]. The 2014 Central Brain Tumour Registry of the United States report found that the overall 10-year survival rate for patients with an ependymoma of any grade is 79.2% [7]. In general, the survival rate peaks for those aged 20–44 years (89.7%) and decreases with increasing age at diagnosis (66% for those aged 0–19 years; 28.1% in the over 75 s) [3].

Incorrect initial histological diagnosis [8], and a degree of subjectivity as to what the correct histological diagnosis are problems [9, 10], which confound the large retrospective studies on ependymomas. Accurate diagnostic neuropathological staining remains of vital importance from a research point of view, as only marrying this with the new molecular categorization will allow us to answer the question as to whether classical histology can have a meaningful impact on prognostication and treatment strategy of ependymomas.

In this paper we aim to correlate the characteristics of Grade II ependymomas with three immunohistochemical markers:

- GFAP—an intermediate filament protein that is expressed by numerous cell types of the central nervous system including astrocytes and ependymal cells during development.
- EMA—a transmembrane mucin widely expressed on epithelial cells, meningothelial cells and ependymal cells and is traditionally a helpful diagnostic marker for carcinoma, meningioma and ependymoma.
- Ki-67—a cell cycle marker for proliferation; it is an excellent marker to determine the growth rate of a given cell population.

The current literature suggests that these markers may have an impact on classification, prognostication and outcome in ependymomas. Here we investigated how the biomarker status of the tumours in our patient population correlated with the location, morphology and behaviour of the adult WHO grade II ependymomas. We looked at all adult patients admitted for surgery of an intracranial lesion with a post-operative histological diagnosis of WHO grade II ependymoma at our neurosurgical unit between January 2008 and January 2018. Histopathological and molecular data were collected from neuropathological reports.

Immunostaining was performed using a Bond-max™ automated staining system (Leica Biosystems, Newcastle, UK). The immunolabelling was visualized using the Bond Polymer Refine Detection kit (DS9800, Leica Biosystems, Newcastle, UK). The negative controls were treated identically except that primary antibody was omitted. All antibodies were obtained from DAKO, Golstrup, DK; GFAP (Z0334, pretreatment H1(5), 1:100 dilution), EMA (E29, pretreatment H1(10), 1:150 dilution), Ki67 (M7240, pretreatment H2(20), 1:100 dilution). Pretreatment codes: H1(5) = Epitope Retrieval Solution 1 (ER1) for 5 minutes; H1(10) = pitope Retrieval Solution 1 (ER1) for 10 minutes; H2(20) = Epitope Retrieval Solution 2 (ER2) for 20 minutes.

The extent of resection was calculated based on a senior neuroradiologist review of post-operative magnetic resonance imaging (24–72 hours). The histological results of the EMA and GFAP results were classified as follows: null—0, weak—1 and strong—2. The Ki67 was analysed based on the percentage of the positive results.

DNA methylation array was performed in a subgroup of five cases on extracted DNA from formalin fixed paraffin embedded tissue by Illumina Infinium Human BeadChip (850K and EPIC; Illumina Inc, San Diego, California). The result were analysed according to the dkfz-Heidelberg classifier (v11b4).

This information was obtained retrospectively after the patients were completely treated according to the initial diagnosis.

The statistical analysis was performed with STATA 13.0® software. The survival and time to recurrence analysis were performed using Kaplan–Meier Curves and Cox Hazard Ratio statistics. To verify the impact of the demographics and location of the tumour on the molecular marker ki67, linear and multilinear regression analysis was performed. The similar analysis for EMA and GFAP was performed using ordered logistic regression. A *P-*value < 0.05 was considered significant for the statistical analysis.

## CASE SERIES

Seventeen patients were included (nine males, eight females, mean age of 51.29 ± 3.91 years old). Seven patients had supratentorial (ST) tumours and 10 patients had infratentorial (IT) tumours. In 10 patients tumours were located on the midline. Fifty-three percent of patients (9/17) had a single procedure. Subtotal resection was the most frequent procedure at the time of the first surgery (58.82% of patients; 42.86% ST and 70.0% IT). At the time of the second surgery, 60% of the patients had GTR. Only one patient had three interventions. All patients were diagnosed with WHO grade II ependymoma (as per inclusion criteria, prior to methylation profile data). All patients were GFAP positive (15 strongly positive and two weakly positive) and EMA positive (13 strongly positive and four weakly positive). The mean Ki67 was 5.19 ± 1.44. Seven patients had radiotherapy (four ST and four IT); five had 54Gy and two had 59.4Gy. Two patients had chemotherapy, both in a supratentorial location. Four patients had hydrocephalus requiring CSF diversion after surgery in the form of ventriculo-peritoneal shunt (one after a failed attempt of a ventriculostomy). This series has a mean follow-up period of 67.76 ± 10.53 months (Table 1).

**Figure 1:**
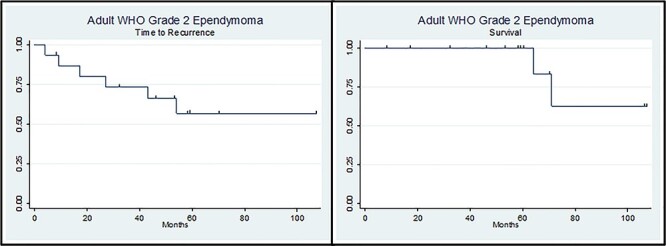
Kaplan–Meier curves of ‘Time to Recurrence’ (left side) and ‘Survival’ (right side).

In the course of 10 years that the study ran, there were three deaths (one ST and two IT, two females and one male). Eight patients had recurrent disease (mean time to recurrence was 44.57 ± 20.08 months); two of them with progression in the WHO grading. Both univariate and multivariate models for risk factors for tumour progression (age, gender, location, extent of resection, chemotherapy, radiotherapy and immunohistochemical markers (GFAP, EMA and Ki67) showed no statistical significant relation with tumour progression (Fig. 1; Tables 2 and 3).

**Table 2:** Univariate analysis of the risk factors for tumour recurrence

	HR	95% CI	*P-*value
Age	1.04 ± 0.04	[0.96–1.12]	0.314
Gender	6.99 ± 7.72	[0.80–60-91]	0.078
EOR first surgery	1.34 ± 0.96	[0.33–5.47]	0.676
Location	2.02 ± 1.76	[0.37–11.17]	0.418
Chemotherapy	2.93 ± 3.29	[0.33–26.37]	0.337
Radiotherapy	0.85 ± 0.74	[0.15–4.68]	0.855
GFAP	0.92 ± 1.0	[0.11–7.90]	0.938
EMA	0.31 ± 0.25	[0.06–1.55]	0.154
Ki67	0.89 ± 0.14	[0.65–1.21]	0.453

EOR, extent of resection; GFAP, glial fibrillary acidic protein; EMA, epithelial membrane antigen.

**Table 3:** Multivariate analysis of the risk factors for tumour recurrence

	HR	95% CI	*P-*value
Age	0.95 ± 0.06	[0.85–1.07]	0.449
Gender	93.68 ± 9.91	[0.09–9.53]	0.149
EOR first surgery	0.005 ± 0.26	[0.002–112.06]	0.301
Location	174.71 ± 909.46	[0.006–47.11]	0.321
Radiotherapy	72379.72 ± 725.7	[0.002–245.12]	0.264
EMA	0.0003 ± 0.002	[0.0759–134.50]	0.223
Ki67	3.23 ± 3.16	[0.471–22.005]	0.231

EOR, extent of resection; GFAP, glial fibrillary acidic protein; EMA, epithelial membrane antigen.

**Figure 2:**
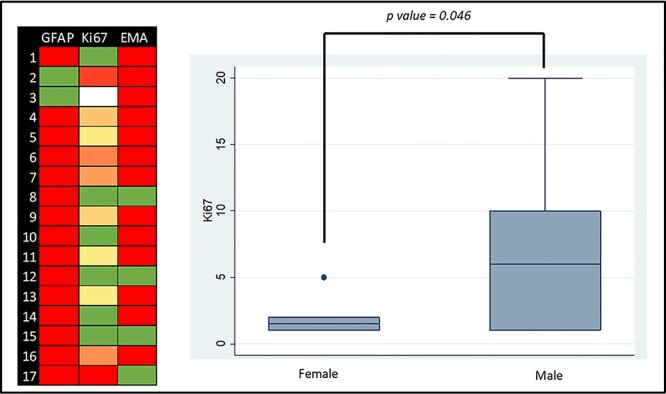
Molecular markers graphic representation: Left side—colour map of GFAP, EMA and Ki67 (green is the lowest value and red the highest value; white is absent information); right side—box plot graph of the distribution of the Ki67 expression according to gender.

The relationship between the demographic variables and tumour location with the molecular markers was studied. In the unadjusted analysis, a statistically significant higher Ki67 was found in the male subgroup (*P* = 0.046). EMA and GFAP were not statistically significantly related with gender, age or location of the tumour (*P* > 0.05) (Fig. 2; Table 4).

**Table 4:** Univariate analysis of the impact of demographic factors and location of the tumour on the molecular markers

GFAP			
	Coef.	95% CI	*P-*value
Age	0.06 ± 0.05	[−0.04 to −0.16]	0.236
Gender	−0.13 ± 1.51	[−3.08 to −2.81]	0.929
Location	18.69 ± 5708.0	[−11168.78 to −11206.15]	0.997
EMA			
	Coef.	95% CI	*P-*value
Age	−0.008 ± 0.04	[−0.081 to −0.066]	0.831
Gender	−0.15 ± 1.14	[−2.40 to −2.09]	0.893
Location	−17.89 ± 3557.55	[−6990.6 to −6954.8]	0.996
Ki67			
	Coef.	95% CI	*P-*value
Age	−0.05 ± 0.06	[−0.176 to −0.069]	0.363
Gender	−3.42 ± 1.54	[−6.786 to −0.071]	0.046
Location	−2.83 ± 1.66	[−6.500 to −0.783]	0.114

GFAP, glial fibrillary acidic protein; EMA, epithelial membrane antigen.

A model for the impact of the demographic—age and gender and anatomical characteristics—location of the tumour on each of the immunohistochemical markers was performed and Ki67 remained statistically significantly higher in the male gender (*P* = 0.043) (Table 5).

**Table 5:** Multivariate analysis of the impact of demographic factors and location of the tumour on the molecular markers

GFAP			
	Coef.	95%CI	*P-*value
Age	0.19 ± 0.15	[−0.113 to −0.484]	0.224
Gender	−3.45 ± 4.17	[−11.64 to −4.73]	0.409
Location	21.78 ± 4614.45	[−9022.381 to −9065.952]	0.996
EMA			
	Coef.	95%CI	*P-*value
Age	0.002 ± 0.039	[−0.074 to −0.078]	0.958
Gender	−0.683 ± 1.337	[−3.304 to −1.938]	0.610
Location	−17.518 ± 2711.8	[−5332.6 to −5297.5]	0.995
Ki67			
	Coef.	95%CI	*P-*value
Age	−0.019 ± 0.047	[−0.124 to −0.086]	0.694
Gender	−3.336 ± 1.443	[−6.551 to −0.122]	0.043
Location	−2.707 ± 1.473	[−5.988 to −0.574]	0.096

GFAP, glial fibrillary acidic protein; EMA, epithelial membrane antigen.

Retrospectively, methylation data was obtained for five patients, being responsible for change in the WHO grading in 3/5 (60%) of cases, two of which were based on the original histological diagnosis and one at the time of recurrence. Two patients remained with the same diagnosis (WHO grade III at the time of recurrence), one patient was upgraded to WHO grade III anaplastic ependymoma and two posterior fossa ependymoma had methylation defined subependymoma.

A sensitivity analysis after inclusion of the methylation data was performed excluding both patients with reviewed methylation-based diagnosis of subependymoma (the other three patients were included as the methylation was based on the samples at the time of recurrence). There were no differences in the time to recurrence and survival analysis. The relationship between male Ki67 and male gender was maintained for both the unadjusted and adjusted analysis with lower *P* values (*P* = 0.030 and *P* = 0.039, respectively).

## DISCUSSION

There is a growing acceptance that genetic and epigenetic profiles are more useful in prognostication than clinical and radiological features or traditional tumour grading on routine neuropathological stains in ependymomas and other CNS tumours [11, 12]. This is corroborated by this retrospective analysis of 17 adult WHO grade II intracranial ependymomas in which age, gender, location, extent of resection, chemotherapy, radiotherapy and immunohistochemical markers could not explain the heterogeneity in survival, recurrence or disease evolution.

However, we do not believe that the recent move of the neuro-oncology community towards the new ependymoma classification based on methylation profile should signal the discarding of decades worth of data with classic neuropathological stains. Current weaknesses such as inter-user variability, intra-tumoural heterogeneity and inability to prognosticate, may be overcome by the development of more sophisticated technologies, including machine learning, and incorporating molecular information as an extra layer of knowledge as is the case with the latest WHO 2016 classification. After all the histological categorization of tumours has for over a century provided the framework for how we understand the origins of a lesion and on several fronts, there is still room for the field to improve [11].

We found Ki67 to be higher in male gender of WHO grade II tumours. A suggestion for the biological rationale behind this finding could include a hormonally mediated effect on the protein expression. Initial support for a hormonally mediated effect is found in Stakišaitis *et al.*’s animal study on Ki67 expression in adenocarcinoma. In adenocarcinomas, the Ki67 index in urethane-treated gonad-intact males was significantly higher than in females and gonadectomized mice of both sexes [13]. Although gender and Ki67 expression were not shown to have an impact on survival in this study, they still provide worthwhile areas for future research as they may be avenues to inform sex and location specific approaches to treatment, including different adjuvant treatments. GFAP, EMA and Ki-67 have been shown in some studies to have associations with certain traditional groups of ependymomas. GFAP has been associated with tancytic [14] and giant cell [15] ependymomas, EMA with anaplastic [16] and giant cell [15] ependymoma and a Ki-67 index <10% with increased progression free survival of supratentorial ependymomas [17]. Xi *et al*’s single-centre retrospective analysis of 69 spinal and cranial ependymal tumours found that whilst location, necrosis, mitosis and Ki67 index were related to prognosis, only Ki67 index was found to be an independent prognostic factor for survival [6].

In this study, even though the DNA methylation data were not available systematically, it provided help to overcome diagnostic uncertainties retrospectively. The sensitivity analysis performed after exclusion of the two patients reclassified as metyhylation-defined subependymomas, further confirm with lower *P values* the findings pre-methylation data. This supports the methylation as a step further for categorization refinement.

Some limitations are worth mention in this study; the sample size is small and larger studies with better power will be required to verify the findings and systematically availability of DNA methylation data will be required to further improve the robustness and quality of these results. Even though the results were adjusted for confounding, the small sample size limit the application of this adjustment. Nonetheless, within these limitations the data presented here arise from a homogenous cohort, as per the most recent WHO classification system, with all patients included in this pilot study being adults, treated in a single centre and under the care of the same multidisciplinary neuro-oncology team. Larger series are required to further confirm the hypothesizes raised by this work of Ki67 being a sex-specific proliferation marker in adult WHO Grade II ependymomas.

## CONCLUSION

The outcome of adult patients with intracranial grade II ependymomas is not associated with clinical and immunohistochemical markers. Ki67 is a potential sex-specific, higher expression in male gender, marker in this patient group.
